# Cytoprotection Concepts for Ischemic Stroke in the Recanalization Era

**DOI:** 10.1002/advs.202517043

**Published:** 2026-02-18

**Authors:** Johannes Boltze, Marc Fisher

**Affiliations:** ^1^ University of Warwick School of Life Sciences Coventry UK; ^2^ Department of Neurology Beth Israel Deaconess Medical Center Harvard Medical School Boston USA

**Keywords:** brain imaging, clinical trial, cytoprotection, ischemic stroke, neuroprotection, recanalization, translational research

## Abstract

Recanalization therapies for ischemic stroke, in particular endovascular thrombectomy, have revolutionized acute stroke management. Cytoprotective approaches were unsuccessfully tested in the pre‐recanalization era but have seen a renaissance in translational research and early clinical trials as a potential intervention to augment the impact of recanalization therapies. The new clinical trial approaches in which cytoprotective therapies are now being applied require refinement of cytoprotective application strategies. This has a profound impact on both preclinical translational and clinical research. This review summarizes current cytoprotection concepts and explains their rationale based on ischemic stroke pathophysiology and provides an overview of cytoprotection approaches currently under clinical assessment. Preclinical assessment of novel cytoprotective paradigms will require advanced in vivo testing in models resembling human stroke patients as much as possible. The review therefore also describes ways to improve preclinical and translational research with respect to comorbidities and other aspects impacting stroke pathophysiology. Moreover, the role of modern brain imaging approaches is discussed including their use as potential biomarkers or patient selection tools. The review further provides detailed considerations of novel clinical trial design features for cytoprotection trials in the context of recanalization therapies and provides an outlook on potential future research approaches.

## Graphical abstract

This review provides detailed information on modern cytoprotective strategies for ischemic stroke in the context of recanalization therapies. It derives their rationale based on contemporary knowledge of stroke pathophysiology and discusses the role of advanced imaging approaches as well as concepts for novel clinical trial designs. It further describes options for improved preclinical and translational assessments of cytoprotective approaches.

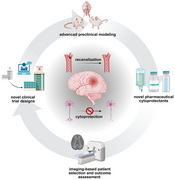

## 1 An introduction to Cytoprotection Concepts in the Recanalization Era

**FIGURE 1:**
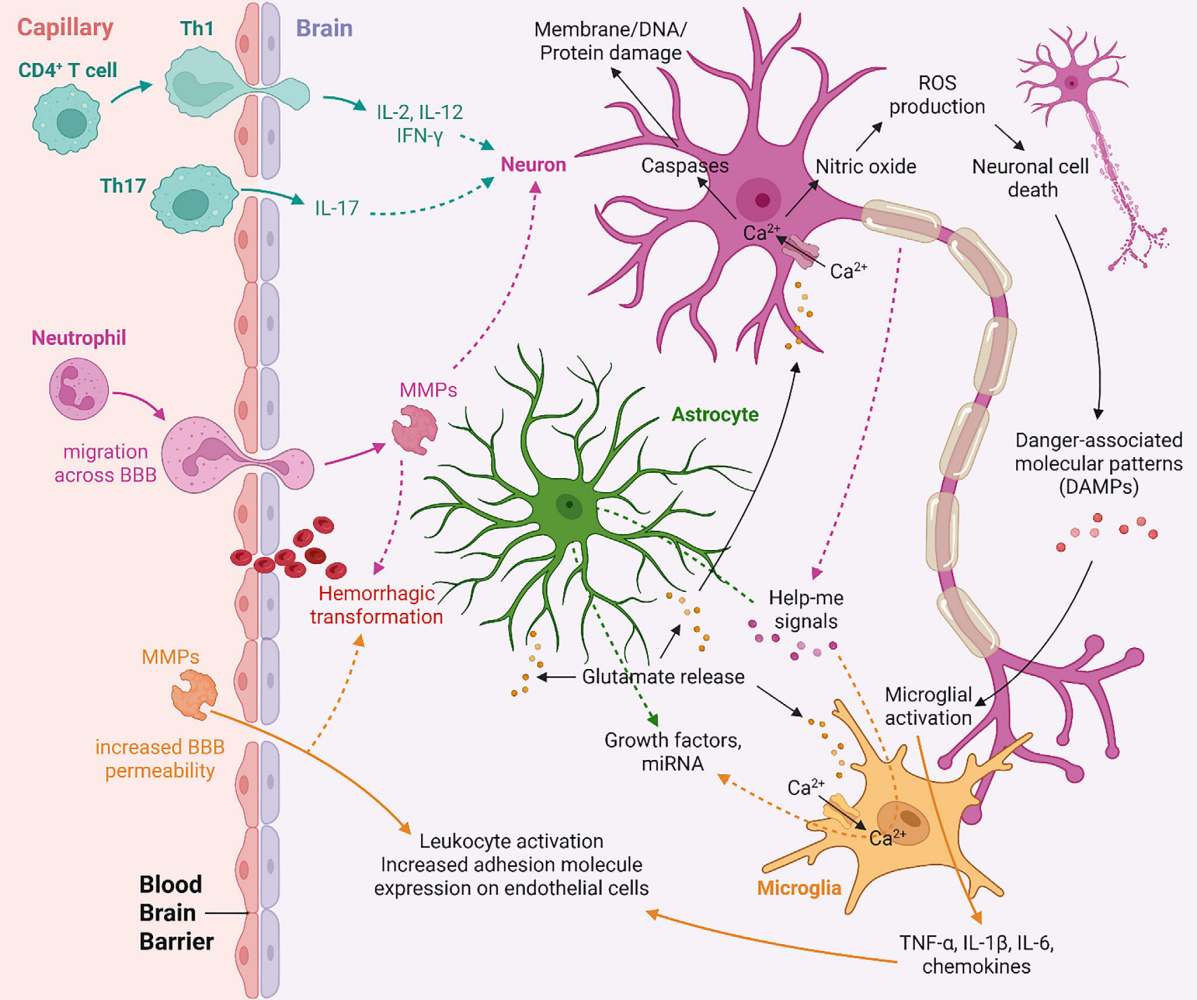
The ischemic cascade. Occlusion of a brain‐supplying artery and subsequent deprivation of oxygen and glucose to brain tissue initiates numerous deleterious effects on cells in affected brain regions. These effects are collectively termed the ischemic cascade and involve direct cell damage, excitotoxicity, peripheral and central immune reactions, oxidative stress, activation of cell death mechanisms, and hemorrhagic transformation. Individual components of the ischemic cascade appear at different times after stroke onset. For instance, excitotoxicity appears within minutes after ischemia onset (i.e., in the hyperacute stage of stroke) whereas peripheral immune responses may take several hours to days (i.e., in the acute to subacute stroke stages). Thus, the ischemic cascade has both spatial and temporal aspects, and its individual elements can be targeted by cytoprotective approaches. Non‐standard abbreviations: BBB: blood‐brain barrier, CD: cluster of differentiation, IFN‐γ: interferon gamma, IL: interleukin, MMP: matrix metalloproteinase, ROS: reactive oxygen species, Th: T helper (cell), TNF‐α: tumor necrosis factor alpha. The figure was created with BioRender.

The use of drugs to protect ischemic brain tissue in stroke patients from evolving to irreversible injury, i.e. infarction has a long and disappointing history. Previously, this therapeutic approach was called neuroprotection but since not only neurons are the therapeutic target the more appropriate term, cytoprotection, is now widely employed. Currently, the conceived target for cytoprotection is the neurovascular unit consisting of multiple cell types including neurons, astrocytes, endothelial cells, pericytes and other glial cells [1]. The concept underlying cytoprotection is that deprivation of oxygen and glucose to brain tissue initiates numerous deleterious effects within the affected cells, termed the ischemic cascade (Figure 1) [2]. Additionally, a detrimental inflammatory response occurs which is characterized by the recruitment of different white blood cells from the peripheral circulation. Apoptotic and other cell death pathways are activated and contribute to the death of brain cells. Some components of the ischemic cascade such as glutamate toxicity are initiated early after the onset of ischemia while others such as apoptotic cell death pathways become more prominent later. The evolution of ischemic injury leading to cell death and tissue infarction is influenced by the degree of blood flow decline with regions of very low or absent blood becoming irreversibly injured more rapidly than brain regions with more modest reductions in blood flow [3]. Metabolic factors such as glucose levels, blood oxygenation and pH can also affect the evolution of ischemic tissue toward infarction. Interestingly, recent research suggests that different elements of the neurovascular unit show different vulnerability to ischemic stress. While neurons are the most susceptible, endothelial cells and pericytes show moderate susceptibility, and astrocytes are the most resilient against ischemic stress [4].

The variable evolution of ischemic tissue injury to irreversibility has led to the concept of the ischemic core and the ischemic penumbra. The ischemic core is the hypoperfused brain region that has already progressed to irreversible injury, i.e. infarction [5]. The ischemic penumbra is the hypoperfused brain region that is not yet irreversibly injured and is potentially salvageable with timely intervention. The extent and evolution of the ischemic penumbra is quite variable among individual ischemic stroke patients. The most important factor affecting the extent and evolution of the ischemic penumbra is residual cerebral blood flow (CBF) that is influenced by the extent of collateral blood supply through alternate channels not directly impacted by the vessel occlusion precipitating the ischemic event [6]. Stroke patients in whom the progression of the ischemic penumbra into the ischemic core is rapid are termed fast progressors, while those patients with a less rapid evolution are termed slow progressors [7]. An intermediate progression group has also been identified.

With the now widespread availability of recanalization by thrombolysis or endovascular thrombectomy (EVT), protecting the penumbra becomes an attractive therapeutic goal in acute ischemic stroke management. Cytoprotection trials conducted in the pre‐recanalization era had to achieve long‐lasting protection, at least to the point of spontaneous clot dissolution and reperfusion, to be able to show a meaningful therapeutic impact. This may be difficult to achieve and consequently, these previous trials failed to show a positive therapeutic effect. EVT is particularly effective in opening occluded proximal blood vessels. Newer cytoprotective drugs are therefore being evaluated in combination with successful recanalization, attempting to augment the impact of recanalization therapies [8]. For instance, this may be achieved by transiently slowing down or even stopping penumbral tissue from evolving into infarction until recanalization is performed [9]. This would preserve a larger volume of salvageable penumbral tissue and thus potentially result in smaller volumes of infarcted tissue after successful recanalization. Achieving transient cytoprotection may be more realistic compared to long‐lasting cytoprotection which would be required in scenarios of delayed spontaneous or no recanalization. We have therefore entered a new era, and it is appropriate to reassess the potential for cytoprotection as a therapeutic approach in combination with EVT and/or thrombolysis for acute ischemic stroke.

Appropriate trial design and diagnostic tools used must be carefully considered when planning clinical trials assessing transient cytoprotection approaches. These trials should also be based on robust pathomechanistic knowledge obtained in basic science and translational research.

## 2 An Overview of Pharmaceutical Cytoprotection Strategies Under Current Investigation

Cytoprotection strategies can address different pathophysiological mechanisms, primarily depending on the time after stroke onset at which they are applied. In the hyperacute stage, i.e., in the prehospital phase or during transfer from a community hospital to a tertiary stroke center, slowing down infarct growth would be a valuable strategy. To achieve this, acute mechanisms of neuronal cell death should be targeted. The recent FRONTIER trial is an example for such an approach [10]. Nerinetide, an eicosapeptide countering glutamate excitotoxic signaling and neuronal nitric oxide production by interacting with post‐synaptic density protein 95, was used with a median time of 64 min after stroke onset to treatment initiation in a prehospital setting. The FRONTIER trial provided preliminary evidence for efficacy in those patients who later underwent recanalization therapy. However, these results must be confirmed in future and larger studies due to limitations in trial design being unavoidable in a study assessing a prehospital intervention. For instance, some baseline data could only be obtained after treatment was already initiated. Cytoprotection therapies that are applied during or immediately after recanalization, i.e., in the acute stage of stroke, may target ischemia‐reperfusion injury (IRI) which is characterized by oxidative tissue stress and subsequent inflammation due to swiftly increasing oxygen levels in reperfused brain regions [11]. Cytoprotective approaches may even offer benefits in late subacute or chronic stroke stages by preventing secondary neuronal cell death due to delayed neuroinflammation in areas deprived from signaling input due to the loss of connections that emerged from the lesioned area [12, 13]. The advent of recanalization therapies and re‐thinking cytoprotective concepts has led to a renaissance of clinical cytoprotection trials. Table 1 provides an overview of cytoprotective agents under current or recent clinical investigation as well as details about their proposed mode of action and potential applicability in different stroke stages. Some studies suggest that the application of next‐generation cytoprotectants such as edaravone‐ dexborneol may even contribute to favorable functional outcome in patients not receiving recanalization [14, 15], although these findings require further confirmation.

**TABLE 1:** Cytoprotective substances under recent or current clinical investigation.

Cytoprotectant	Type	Proposed effect	Trial phase	Stroke stages applied in	Safety	Efficacy	Other aspects	Refs.
3K3A‐APC	pleiotropic PAR1 agonist	neuroprotection, vasculoprotection	2	acute	yes	uncertain*	used with recanalization to reduce ICH rates	Lyden et al., 2019 [16]
Afamelanotide	α‐melanocyte stimulating hormone analogue	neuroprotection, anti‐inflammation	1 and 2	acute	yes	not yet shown		Stanislaus et al., 2023 [17]
ApTOLL	TLR4 antagonist	anti‐inflammation	1 and 2	acute	yes	preliminary evidence		Hernández‐Jiménez et al., 2023 [18]
AST‐004	partial adenosine A1R/A3R agonist	neuroprotection, glioprotection (?)	1	acute	yes	not yet shown		Manna et al., 2024 [19]
Cerebrolysin	porcine neuropeptide mix	unclear / diverse, pro‐regenerative (?)	2	acute into subacute (daily infusions for 14 days)	yes	uncertain	also used with tPA to reduce ICH rates	Khasanova et al., 2023 [20] Poliakovic et al., 2021 [21]
Citicoline	cell membrane stabilizer	cytoprotection, anti‐inflammation, anti‐excitotoxicity (?)	2	acute into subacute (daily infusions for 3 days + oral for another 39 days)	yes	no		Argarwal et al., 2022 [22]
Dl‐3‐n‐butylphthalide	synthetic version of a compound found in celery oil	anti‐inflammation (?), anti‐apoptosis (?), anti‐oxidation (?)	2 and 3	acute into subacute (daily infusions for 14 days +oral for another 76 days)	yes	preliminary evidence	specifically investigated for use with recanalization	Chen et al., 2025 [23] Wang et al., 2023 [24] Luo et al., 2019 [25]
edaravone‐ dexborneol	composite molecule	anti‐oxidation	2 and 3	acute into subacute (daily infusions for 12 or 14 days)	yes	uncertain		Chen et al., 2025 [26] Ma et al., 2025 [14] Fu et al., 2024 [15] Xu et al., 2021 [27] Xu et al., 2019 [28]
GD‐11	edaravone‐derivative	anti‐apoptosis, anti‐oxidation	2	acute into subacute (daily infusions for 10 days)	yes	no	designed for better BBB crossing, converts in edaravone in plasma	Zhang et al., 2025 [29]
glyceryl trinitrate	nitric oxide donor	vasodilation, anti‐oxidation	2	acute	yes	no		Cai et al., 2024 [30] Cheng et al., 2023 [31]
methylprednisolone	corticosteroid	anti‐inflammation anti‐oxidation	3	acute into subacute (daily infusions for 3 days)	yes	no, but better safety outcome	specifically investigated for use with EVT	Yang et al., 2024 [32]
nelonemdaz	NMDAR blocker radical scavenger	anti‐excitotoxicity anti‐oxidation	3	acute into subacute (daily infusions for 5 days)	yes	no	specifically investigated for use with EVT	Lee et al., 2025 [33] Hong et al., 2022 [34]
nerinetide	eicosapeptide	anti‐excitotoxicity anti‐oxidation	2 and 3	hyperacute (prehospital), acute	yes	preliminary evidence (hyperacute), no (acute)		Christenson et al., 2025 [10] Hill et al., 2025 [35]
odatroltide	P‐selectin inhibitor	anti‐oxidation, anti‐inflammation, believed to improve microcirculatory blood flow	2	acute	yes	no	specifically investigated for use with recanalization	Chao et al., 2024 [36]
otaplimastat	matrix metalloprotease inhibitor	edema reduction prevention of hemorrhagic transformation	2	acute into subacute (daily infusions for 3 days)	uncertain	no		Kim et al., 2020 [37]
RNS60	oxygen‐enhanced saline	cytoprotection anti‐inflammation	2	acute	yes	preliminary evidence (reduced infarct growth in one group)		Ghosh et al., 2025 [38]

^*^3K3A‐APC efficacy was assessed as a secondary endpoint in this study. Based on reported vasculoprotective effects, rates and volumes of intracranial hemorrhages (ICHs) was investigated. While there was a statistically significant difference in the previous, no difference was found for the latter (p = 0.066). Efficacy was therefore rated as “uncertain”. The reduction of ICH rats is both an efficacy indicator (as a presumed consequence of vasculoprotection effects) and a safety benefit.

Given the complexity of the ischemic cascade (Figure 1) and stroke‐related pathophysiological mechanisms in exerting an impact at different times after stroke onset, it is unlikely that targeting a single pathological mechanism will be sufficient for robust cytoprotection [39]. More effective approaches can be envisioned. One option would be to use single molecules targeting several elements of the ischemic cascade. An example is DL‐3‐n‐butylphthalide which is believed to exert anti‐apoptotic, anti‐oxidative and anti‐inflammatory effects. The molecule has been recently investigated as a cytoprotectant in patients receiving recanalization therapy in China [23, 24]. Importantly, many elements of the ischemic cascade such as apoptosis, neuroinflammation and oxidative stress are interdependent. Thus, claiming that a molecule targets different elements of the ischemic cascade requires explicit proof that the molecule indeed acts on these elements independently. The causal connection of elements of the ischemic cascade may also cause overlap regarding the proposed mechanism of action of a cytoprotectant. For instance, cytoprotectants mitigating inflammation may also be classified as neuroprotective because reduced inflammation may mitigate secondary neuronal damage. A precise explanation of the confirmed modes of action by a particular molecule in the literature would help to overcome such terminological impreciseness.

Alternatively, a combination of molecules each addressing different targets in the ischemic cascade could be used. Future clinical trials may therefore focus on cytoprotective strategies addressing multiple targets in one stroke stage or addressing at least one target at different stroke stages. This will likely require the use of multiple drugs. In case the intervention is effective, effect sizes observed in those trials may be larger. This might facilitate proof of efficacy studies because smaller effect sizes of a cytoprotective intervention may be statistically “masked” by the substantial therapeutic impact of the recanalization therapy. Conversely, challenges associated with multi‐drug cytoprotective approaches can also be anticipated. First, there may be the necessity to include cytoprotectants developed by competing companies that may not necessarily agree to collaborate for such trials. Second, the individual contribution of drugs used in combination to increase the therapeutic effect will be difficult to quantify. Third, the exact cause of potential detrimental effects would be much harder to detect in such combination trials. Platform trials may be an option to deal with such issues but have only recently been introduced in clinical stroke research [40].

Non‐pharmacological cytoprotective approaches being investigated in parallel to pharmacological cytoprotection include the use of medical devices [41], remote ischemic conditioning [42, 43], physical interventions such as normobaric hyperoxia [44] or selective hypothermia [45]. Non‐pharmacological cytoprotective approaches rely on diverse therapeutic mechanisms, often being different from those of pharmacological cytoprotective approaches. Such approaches have been reviewed elsewhere [46]. Therapeutic mechanisms exerted by non‐pharmacological and pharmacological cytoprotectants can overlap or show synergistic effects. Thus, clinical trials combining both approaches should be considered in the future.

It is important to note that some cytoprotective approaches have shown no or uncertain efficacy in recent trials (Table 1). The reasons for that are numerous. First, clinical trial results may strongly suggest that a cytoprotectant candidate is indeed not efficacious. In this case, it might be withdrawn from further development. Second, cytoprotectants may not meet efficacy outcomes due to design specifications or suboptimal application paradigms in some clinical trials. In this case, alternative application scenarios may be discussed. Third, early phase clinical trials (phase 1 and 2) typically focus on safety, feasibility and tolerability endpoints and are therefore underpowered to meet any secondary efficacy endpoints. Proof of efficacy will require larger and properly powered clinical trials to investigate efficacy endpoints. Fourth, we defined efficacy as uncertain when there was divergent information on meeting efficacy outcomes from different clinical trials, or in case some but not all efficacy endpoints were met in a single clinical trial. Downstream clinical research on those cytoprotective candidates may rely on a refined application regimen, the selection of patients who may benefit more likely from the intervention, or alternative efficacy endpoints. This may require a detailed review of design differences between clinical trials. An alternative approach to select promising cytoprotection candidates before entering clinical trials is to conduct confirmatory multicenter preclinical randomized and controlled studies such as in the Stroke Preclinical Assessment Network (SPAN) [47].

## 3 Stroke Modeling and Increased External Validity in Preclinical and Translational Cytoprotection Research

The investigation of cytoprotective drugs in preclinical stroke models is important for informed decisions on promising application strategies in clinical trials. Models of transient cerebral vessel occlusion are available. Noteworthy, prior animal studies assessing cytoprotective agents have typically demonstrated better effectiveness with transient vessel occlusion as compared to permanent vessel occlusion [48]. Most animal models focus on the occlusion of the middle cerebral artery (MCA) because human stroke often manifests in the MCA supply territory for anatomical and hemodynamic reasons. The classical filament model for transient MCA occlusion (tMCAO) is a well‐established and effective model that can be used in rodent species to mimic endovascular thrombectomy in large proximal vessels [49]. The common tMCAO filament insertion techniques normally cause additional infarction in the posterior cerebral artery supply territory but refined approaches inducing standardized infarcts only within the MCA supply territory have been developed [50]. There is also the option to use external mechanical pressure on the MCA, for instance by a surgical clip, for tMCAO. Such approaches are well controllable but used less frequently as they require craniectomy. Thromboembolic rodent stroke models are also available. These rely on the deposition of an autologous blood clot in the appropriate intracranial vessel. A major advantage of these models is that recanalization by thrombolysis can be evaluated [51, 52]. However, thromboembolic models tend to be more variable regarding stroke location and size because the final position of the clot is not always fully reproducible. While this higher heterogeneity may require larger sample sizes to detect a certain treatment effect, it also adds to external study validity. This can be important for translational research studies. Other tMCAO models rely on topical or intra‐arterial endothelin‐1, thrombin and ferric chloride applications [53]. Some of these models tend to be more variable regarding stroke outcomes while others are technically demanding or do not allow for controlled recanalization. These models have been proven highly useful for specialized experimental questions such as the development of novel thrombolytics but are rarely employed in studies assessing experimental cytoprotectants. Finally, photothrombotic stroke models are available [54]. Those do not allow for reliable reperfusion and are therefore less relevant when investigating cytoprotective approaches in the context of recanalization. However, photothrombotic stroke models allow induction of infarction in precisely determined cortical areas which might be meaningful when investigating specific effects of cytoprotectants.

External validity of preclinical studies can be increased by using animal stroke models with relevant comorbidities. There is an excellent availability of rodent species exhibiting comorbidities that are relevant for human stroke patients. Typically, these comorbidities do not require induction or can be induced easily and non‐invasively, for instance by providing a specialized diet. One example is hypertension which is among the most relevant clinical stroke comorbidities. Stroke‐prone spontaneously hypertensive rats (SP‐SHR) suffer from severe hypertension that can be further exaggerated by a high‐salt diet. These animals experience spontaneous intracerebral hemorrhage or ischemic strokes which often show hemorrhagic transformation (HT). Spontaneously hypertensive rats (SHR) have a less severe hypertension which does not lead to spontaneous strokes but still causes cerebral blood vessel damage and subsequent cognitive decline during adulthood [55]. Moreover, the collateral capacity of the SHR cerebral circulation is highly limited [56], resulting in larger strokes with a smaller, quickly declining penumbra after MCAO [57]. This is relevant for cytoprotection research because SHR can mimic fast progressors whereas the normotensive Wistar Kyoto (WKY) control strain may resemble slow or intermediate progressors, respectively. SHR also exhibit atrial fibrillation [58]. Stroke models considering further comorbidities are available and include animals with diabetes type I and type II [59, 60], obesity [61], and hyperlipidemia [62]. Importantly, all comorbidities modify stroke impact and outcome, often by contributing to one or more elements of the ischemic cascade (Table 2) and thereby being of high relevance in cytoprotection research. Age and sex are further aspects being relevant in cytoprotection research, and their impact has been reviewed elsewhere [63]. An important aspect of external preclinical study validity is coherence between preclinical and clinical study design. Missing such coherence may contribute to translational failure which has been shown for other experimental stroke treatments [64]. It is particularly important to maintain coherence regarding the analyzed endpoints. From a clinical perspective, the most important primary endpoint is to improve functional outcome. This can be precisely tested preclinically using behavioral tests. Those tests typically assess motor functions, but sensory functions can also be investigated. It is important to use tests which can reliably discriminate between functional compensation and true functional recovery because functional recovery can be mimicked by functional compensation in rodents [65]. Other clinically relevant endpoints are the reduction of post‐stroke cognitive impairment and depression. Although cognitive function and anhedonia (as an approximation of depression) can be assessed in rodent models, such endpoints are often not investigated in preclinical studies. Refining preclinical study protocols in this regard may improve the external validity of preclinical studies.

**TABLE 2:** Common comorbidities in animal stroke models and their impact on the ischemic cascade.

Comorbidity	Induction	Proposed impact	Affected elements of the ischemic cascade	Refs.
Hypertension	spontaneous (genetic) optionally with dietic modification; pharmacological (e.g., angiotensin II); surgical (e.g., renal artery constriction)	Decreased penumbral volume and persistence due to vascular remodeling, modified vascular tone, and decreased collateral capacity	All	Cipolla and Chan, 2020 [56]
		Increased neuroinflammation by microglial activation, further aggravating hypertension	Microglial activation	Shen et al., 2015 [66] Marks et al., 2001 [67]
		Peripheral proinflammatory priming; increased adhesion molecule and chemokine expression, higher number of infiltrating leukocytes	Neutrophil migration across BBB, neutrophil activation	Möller et al., 2015 [68] Santisteban et al., 2015 [69]
Diabetes	spontaneous (genetic), sometimes combined with obesity (Zucker rats); pharmacological (streptozotocin); type 1 and type 2 diabetes models exist	Increased endothelial oxidative stress, impaired vessel relaxation, CBF reduction	Endothelial cell damage and increased BBB permeability	Chrissobolis et al., 2011 [70]
		Increased pro‐inflammatory signaling; increased adhesion molecule expression, higher number of infiltrating leukocytes	Neutrophil migration across BBB, neutrophil activation	Venkat et al., 2017 [71] Ritter et al., 2011 [72]
		Metalloproteinase activation (e.g., MMP‐3/‐9), endothelial permeability and damage	increased HT and edema	Elgebaly et al., 2011 [73]
		Increased neovascularization (new vessels are immature and permeable)	increased HT	Ergul et al., 2014 [74]
Hyperlipidemia	dietary, spontaneous (genetic)	Reduced perfusion due to impaired autoregulation; reduced collateral capacity	All	Hermann et al., 2019 [75] Ayata et al., 2013 [62]
		Increased platelet‐endothelial/leukocyte‐endothelial interaction and focal thrombosis	All	Ishikawa et al., 2004 [76]
		Increased pro‐inflammatory mediator expression and neuroinflammation	Neutrophil migration across BBB, neutrophil activation	Herz et al., 2015 [77] Cao et al., 2015 [78]
Obesity	dietary (overlapping with hyperlipidemia), genetic in combination with diet	Metalloproteinase activation (e.g., MMP‐9), endothelial permeability and damage	Increased HT and edema	Deng et al., 2014 [79]
		Increased pro‐inflammatory mediator expression and neuroinflammation	Unclear, neutrophil activation / migration (?)	Haley et al., 2017 [80]
		Reduced expression of adiponectin which is cytoprotective	Unclear, oxidative stress (?)	Li et al., 2017 [81]

Studies using rodent stroke models can be complemented by experiments in recently emerging large animal stroke models [82]. In large animal species without a rete mirabile such as canines and non‐human primates, endovascular transient vessel occlusion is achieved by inflating a balloon or the catheter‐based deposition of a thrombus at a desired location [83]. The latter approach also allows for recanalization by thrombolysis or thrombectomy [84], closely approximating the situation in human stroke patients. Although the use of large animal models is substantially more expensive and requires dedicated infrastructure as well as specifically trained scientific and support staff, large animal models offer certain additional advantages. All of these can be relevant for the assessment of cytoprotective approaches. First, large animal species have a gyrencephalic brain which has a much larger white matter percentage than rodents. The proportion of white matter in some large animal brains is only slightly lower than that observed in humans [85]. This is relevant for cytoprotective approaches not only targeting gray but also white matter. Second, the larger brain permits the use of clinical imaging equipment without compromising spatial and temporal resolution of the applied imaging protocol. This enables longitudinal imaging studies, for instance assessing the impact of a cytoprotectant on infarct growth in acute stroke [86] or aiding the preclinical development of imaging biomarkers. Hybrid imaging modalities such as magnetic resonance imaging (MRI) combined with positron emission tomography can be used for detailed mechanistic insight in vivo [87], for instance assessing penumbral metabolism or the extent of IRI under a cytoprotective approach. Large animal stroke models can be used to investigate the impact of prehospital cytoprotective interventions in the acute stroke stage by applying sequential brain imaging with high temporal resolution. Such interventions and approaches may be of high clinical relevance but are difficult to investigate in human stroke patients. Third, large animals are typically outbred strains with inter‐individual differences in cerebral collateral capacity. Thus, fast and slow progressors can be identified using clinically relevant imaging approaches such as MRI [88]. Fourth, large animals have a richer behavioral repertoire than rodents [89] enabling detailed long‐term investigation of functional outcomes after a cytoprotective intervention. Fifth, the larger size permits obtaining more frequent and larger samples of blood, cerebrospinal fluid or tissue. This is beneficial to study cytoprotectant pharmacokinetics and ‐dynamics. Finally, large animal models allow the investigation of medical devices and non‐pharmacological cytoprotection strategies, some of which cannot be assessed in rodent stroke models because of size constraints [45]. Due to the infrastructural and financial demands, the use of large animal models is typically restricted to the investigation of aspects that cannot be adequately addressed in rodent models. The Stroke Treatment Academic and Industry Roundtable (STAIR) recommendations encourage studying cytoprotective approaches in gyrencephalic large animal species where appropriate [90]. Comorbidities can develop in large animals, but this usually takes a long time or requires targeted induction. Thus, research on the role of comorbidities in the context of cytoprotective approaches remains a domain of rodent stroke models.

Preclinical studies on cytoprotective strategies should be designed to be either exploratory or confirmatory. Exploratory preclinical studies will create tentative evidence of efficacy in standard animal models, typically in rodents. Exploratory studies can also provide detailed insight into modes of action of the cytoprotective approach. They can further help to generate working hypotheses on translationally relevant aspects such as optimal dosing and timing of the intervention. Preclinical confirmative studies should be adequately powered to show efficacy in animal models approximating the situation of human stroke patients, for instance by including aged animals, mixed‐sex cohorts and animal models with comorbidities relevant to stroke. Where appropriate, confirmatory studies can also be conducted in large animals. Confirmatory studies are typically powered to assess a set of pre‐defined primary endpoints but can include secondary endpoints being assessed with lower statistical power if required. Ideally, they should confirm central elements of pharmacodynamics and pharmacokinetics of the substance being investigated, as well as the most feasible route of administration and dosing. The minimum set of endpoints should be functional outcome and lesion volumetry, ideally in a dynamic, longitudinal setup, i.e., infarct core growth and penumbral decline investigated by serial brain imaging. In case the cytoprotective concept includes the use of biomarkers to identify patients responding best or benefitting most from the intervention, these biomarkers should be assessed in confirmative studies when possible.

## 4 Modern Imaging Approaches in Cytoprotection Trials

**FIGURE 2:**
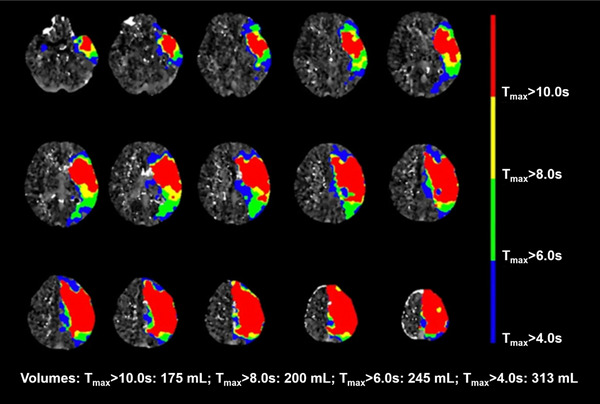
Imaging example of a fast stroke progressor CT perfusion scans of a patient with large volumes of tissue exhibiting high T_max_ values. The hypoperfusion intensity ratio in this patient is 0.71. This patient is very likely a fast stroke progressor who may benefit from cerebroprotective approaches during transfer times.

Fast, slow or intermediate stroke progressors can be identified with advanced imaging techniques such as computed tomography perfusion (CTP) or diffusion/perfusion MRI. These imaging modalities are widely available at larger stroke centers and a commonly obtained as part of the initial evaluation of ischemic stroke patients. CTP, an approach to characterize the rapidity of ischemic core evolution, has been used to identify the percentage of the ischemic region with very low residual CBF in relationship to extent of brain tissue with a more modest reduction in CBF in the ischemic region. This relationship is called the hypoperfusion intensity ratio (HIR). The HIR is defined as the ratio of cerebral tissue volume with a time‐to‐maximum (T_max_) of greater than 10 s divided by the volume of T_max_ greater than 6 s. Patients who have a HIR of at least 40–50% have a high probability of being fast progressors (Figure 2) [91].

The therapeutic target of cytoprotection is to try to salvage as much of the ischemic penumbra as possible to reduce the extent of the final brain infarction [9]. The smaller the final infarction, the more likely the clinical outcome will be better, especially in more eloquent brain regions. In the older clinical trials of cytoprotective agents advanced imaging modalities such as CTP and MRI were not available to select patients more likely to respond to the therapeutic intervention. Additionally, the molecules used in the older clinical trials typically only targeted one component of the ischemic cascade which is now considered a suboptimal approach. Moreover, past cytoprotection trials mostly included patients who did not receive thrombolytic therapy to restore CBF to ischemic brain regions and were performed before the current era of highly effective recanalization therapy by EVT.

**FIGURE 3:**
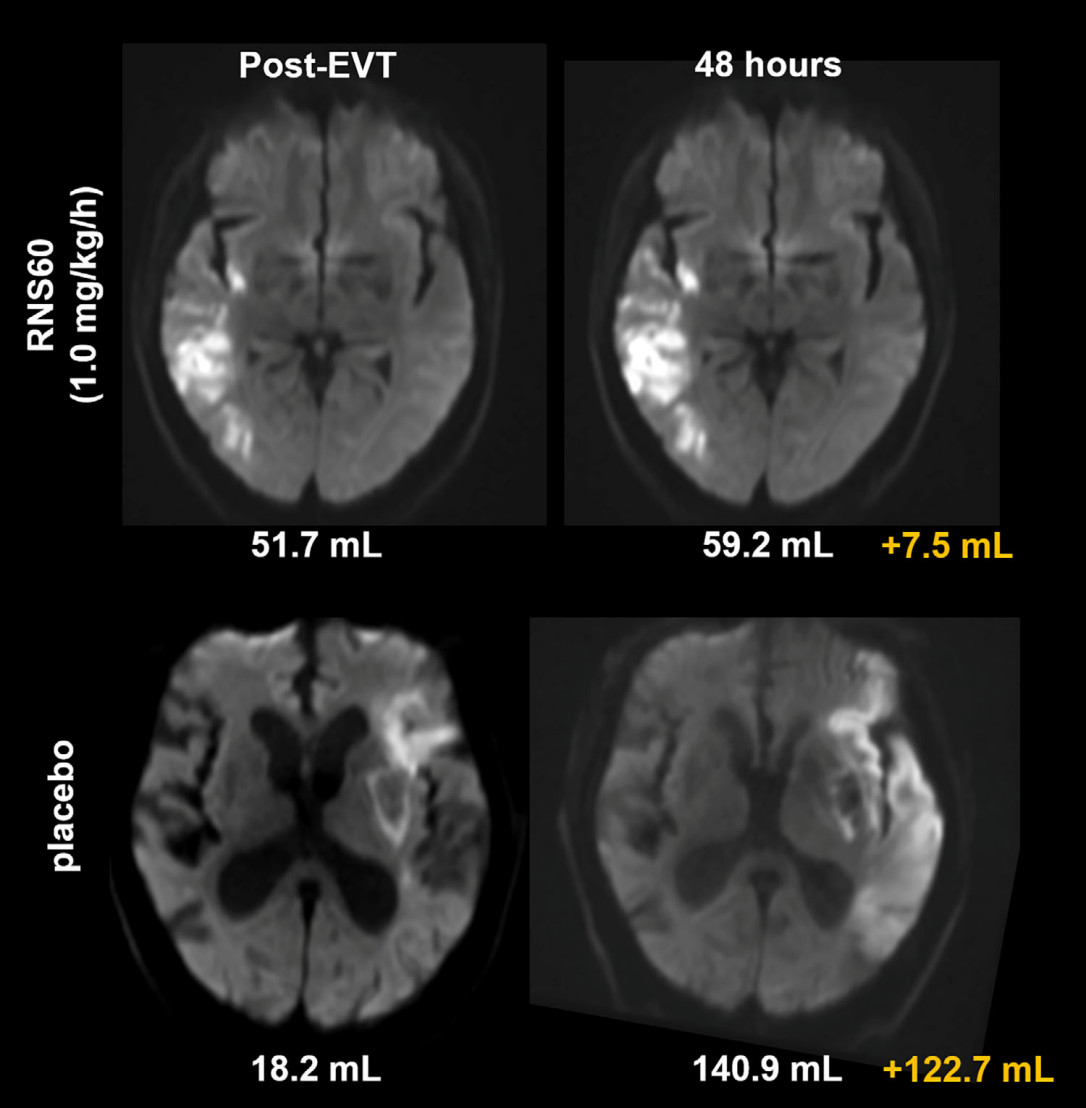
Imaging outcome of a cerebroprotective intervention started during EVT. Diffusion weighted magnetic resonance imaging (DWI) scans from two patients in the RESCUE trial. The patient receiving the verum, RNS60, at the high dose (1.0 mg/kg/h). The treatment was initiated during the EVT procedure and lasted for 48 h. The verum‐treated patient showed only marginal infarct growth in DWI whereas the patient receiving placebo treatment exhibited a dramatic infarct growth. Across the trial, patients treated with high‐dose RNS60 experienced a significantly decreased infarct growth from post‐EVT imaging to 48 h.

Advanced imaging cannot only be used to identify appropriate patients for inclusion in cytoprotection trials but can also be used as a surrogate outcome measure, especially in phase 2 proof of concept trials [92]. In such trials, patients would undergo baseline, pretreatment CTP or MRI to quantify the volume of the ischemic core and penumbra. If they meet prespecified enrollment criteria, they can then be randomized into active drug or placebo groups. CTP can also be utilized to identify ischemic stroke patients more or less likely to respond to cytoprotection to improve trial efficiency by not including patients likely to be uninformative [93]. Follow‐up imaging is then obtained 48–72 h later. The volume of the ischemic core is identified and compared to the baseline ischemic core volume to determine the extent of infarct growth in the treated and control groups. If the treated group has substantially less infarct growth, then there is direct evidence that the investigational cytoprotective drug has cytoprotective effects because it has prevented infarct expansion. Additionally, safety and clinical outcomes are evaluated as in a standard phase 2 clinical trial. Identifying an in vivo cytoprotective effect should reassure trial investigators and sponsors that the cytoprotective drug being studied should be an appropriate candidate for a large, phase 3 clinical trial that is appropriately powered to detect a clinically meaningful treatment effect. Such phase 2 trials with an assessment of infarct growth on imaging have already been conducted and reported [18]. An example of a reduction in infarct growth on MRI from the recently reported trial of RNS60 is provided in Figure 3. For the first time, these trials provide evidence that cytoprotective drugs can salvage ischemic tissue in the ischemic penumbra, leading to smaller final infarct volumes that should translate into better clinical outcomes in larger phase 3 trials. The results of these trials can also potentially be used to provide supportive evidence for regulatory authorities (in addition to clinical outcome‐based phase 3 trials) what should accelerate the approval process.

In addition to such proof‐of‐concept phase 2 trials, imaging could be employed in other trial scenarios. Some cytoprotection development programs are being designed to evaluate the treatment effectiveness of molecules when combined with EVT. If the trial will only initiate therapy after successful EVT, then imaging could be utilized to only enroll patients who have an apparent need for additional treatment. In such a trial, patients would undergo CTP or MRI after EVT to identify an appropriate extent of residual ischemic penumbra and to confirm that the ischemic core is not too large. Follow‐up imaging could also be obtained at 48–72 h after treatment initiation to evaluate the extent of infarct growth as previously described. Another approach to cytoprotection is to evaluate molecules targeting IRI.

Importantly, there is constant progress in brain imaging technologies and their applications. There is also a refinement of brain imaging parameter to increase the amount of information being provided by brain scans while reducing the time required to perform them. These refinements may improve acute stroke management but can also provide deeper insight into pathology and response to therapeutic interventions during follow‐up. Current improvements and important areas for further advancements have been recently identified and are reviewed elsewhere [94]. Moreover, artificial intelligence (A.I.) may improve clinical trial design and workflow, including trials on cytoprotection approaches. For instance, A.I. could be used to extract information from non‐contrast CT images. A well‐trained A.I. may further select patients for enrollment into clinical trials and may perform in‐depth analyses linking imaging and clinical data. The use of A.I. in acute stroke trials has been reviewed in detail elsewhere [95].

## 5 Cytoprotection Clinical Trial Design in the Recanalization Era

Clinical trial considerations and design are significantly different in the current era of highly effective recanalization therapies. Most clinical trials of cytoprotection will occur with one or both of the available reperfusion therapies. Several different clinical trial scenarios can be envisioned. One approach would be to enroll ischemic stroke patients at an outlying hospital or in the ambulance as they are being transported to a tertiary center for EVT. In this clinical trial scenario, patients would be evaluated at the outlying hospital by local staff and undergo imaging at that facility. Employing telemedicine, the clinical trial investigator at the tertiary center can interact with the local staff and assess the imaging studies obtained at the outlying facility to help in deciding if the patient meets the enrollment criteria. The patients can then be randomized into the trial. Study drug treatment will be initiated and then continued during transport to the tertiary center. If CTP can be performed at the outlying hospital, which would be ideal, it can then be compared to a repeat CTP at the tertiary center to assess ischemic lesion growth. Only patients with a minimum transport time of at least 1–2 h should be included and slow progressors excluded because they would be unlikely to have much infarct expansion during transport. The primary efficacy outcome in such a trial would be ischemic core growth, but secondary outcomes would include the percentage of patients who remain good EVT candidates, who are likely to experience good 90‐day clinical outcomes. Safety endpoints would also be assessed because these trials would be appropriate for phase 2.

If CTP is not available at the outlying hospital, a different type of phase 2 trial can be envisioned. In this type of trial, only basic CT imaging would be obtained, a head CT and a CT angiogram to exclude intracerebral hemorrhage (ICH) patients, and to identify those with a large vessel occlusion who would be EVT candidates. Randomization would again occur in the outlying hospital with the help of telemedicine input from the tertiary center. Study drug treatment would be initiated and then continued during transport. The patients would then undergo advanced imaging at the tertiary center to assess ischemic core size. The percentage of patients who remain good EVT candidates because they do not have a large ischemic core size (i.e., <70‐100 mL) would be the primary outcome with secondary outcomes, similar to those suggested previously. Clinical trials that only enroll patients in the ambulance during transport to a tertiary center would not be recommended for phase 2 evaluation of a novel cytoprotective drug because of (i) the lack of imaging in standard ambulances that are not equipped with a CT scanner, (ii) heterogeneity of the patients likely to be evaluated for the clinical trial with transient ischemic attacks, ICH as well as stroke mimics likely to be included, and (iii) sample size considerations.

Another type of cytoprotection clinical trial would be conducted in patients presenting in larger hospitals that are EVT‐capable. Stroke patients would undergo clinical and imaging assessment by the stroke team upon presentation. If they meet the inclusion/exclusion criteria for the trial, randomization would occur that should not slow down the performance of the EVT procedure. Study drug treatment would be started before or during EVT and continued after the procedure if the protocol calls for a prolonged infusion. This type of clinical trial design can be used for both phase 2 and phase 3 trials. In phase 2 trials, the primary outcome would be the assessment of ischemic lesion growth as already has been done in some trials. In phase 3 studies, the primary outcome would be evaluated by the modified Rankin Score (mRS) at day 90 and various approaches to determine clinical efficacy on the mRS have been proposed [96]. Secondary outcome analyses may be included providing more granular assessment or insight into specific functional domains and outcomes not assessed by the mRS. Those may include functional improvements in individual limbs, coordination of the pharyngeal musculature for better swallowing, fatigue, or the speed in which activities can be performed by patients. Results from such a phase 3 clinical trial could then be used for regulatory approval. Other types of clinical trials would be to initiate therapy after successful EVT opening of the occluded large artery. One type of trial would target IRI that occurs after restoration of blood flow. Only patients with good to excellent vessel opening would be included. The type of cytoprotective agent to be used in such a trial would be one that is specifically targeted at one or more of the purported mechanisms of IRI [97]. Randomization and initiation of the study drug would occur shortly after EVT completion. Phase 3 clinical trials targeting IRI will likely require a large sample size to detect a significant clinical benefit because the placebo group will have also undergone EVT and will have a substantial percentage of patients with a favorable clinical outcome.

Another type of clinical trial after EVT can also be envisioned. Some patients undergoing EVT do not have a good clinical outcome despite good to excellent vessel opening. Many potential explanations for this lack of benefit have been proposed [98]. One explanation is that despite good vessel opening some portion of the ischemic region has not been salvaged and remains penumbral, for instance due to the no‐reflow phenomenon of insufficient small vessel reperfusion despite successful large vessel recanalization. Cytoprotection given to such patients after EVT might help to prevent this penumbral region from evolving into infarction. Such a trial would require the performance of advanced imaging with CTP or MRI to identify the extent of the penumbra and core as enrollment criteria. Prespecified volumes of core and penumbra would then lead to inclusion or exclusion. Randomization and initiation of study drug would then occur. Such a scenario could be utilized for both phase 2 and phase 3 trials with an imaging primary endpoint for the former and a clinical endpoint for the latter.

## 6 Conclusions and Outlook

Cytoprotective approaches are currently experiencing a renaissance. With renewed concepts of how to use these drugs and novel clinical trial designs, i.e., applying cytoprotective dugs in the context of recanalization therapies, we are starting to see some early evidence of efficacy. This is encouraging. However, such evidence is still limited and tentative, and the results require confirmation in larger trials. The field may also face the need for further refinement of cytoprotective application strategies. For instance, we still lack a thorough understanding of potential circadian effects [99], and reasons for differences between preclinical and clinical studies are unknown. Cell‐ and tissue‐specific cytoprotection time windows suggest that different cell types, i.e., neurons, astrocytes, endothelial cells and pericytes are vulnerable and rescuable at different times after onset of ischemia [100]. We may even think about situations in which a cytoprotective intervention may benefit one cell type but harm another. Conversely, more detailed knowledge about cell‐ and tissue specific cytoprotection time limits may pave the way to improved or even personalized, temporally and spatially optimized cytoprotection approaches in ischemic stroke. Basic, translational and clinical research will have to go hand in hand to disentangle the complex biological relationships between pathophysiological elements such as lesion volume, stroke progression velocity, time of and from onset, and comorbidity profile with the aim to provide individualized cytoprotective treatments to ischemic stroke patients for maximum efficacy.

## Conflicts of Interest

J.B. is a consultant to Aruna Biomedical, Inc. and TargED Biopharmaceuticals B.V., both developing experimental therapeutics for ischemic stroke. M.F. is a consultant to Simcere Innovation, Inc., Lumosa Therapeutics Co., Ltd., and for the Revalesio Corporation. He also serves on the Data and Safety Monitoring Boards for trials sponsored by Moleac Pte Ltd. and Takeda Pharmaceuticals. However, the authors do not declare a conflict of interest regarding the scientific content of this article.

## Data Availability

The authors have nothing to report.

## References

[1] P. D. Lyden , “Cerebroprotection for Acute Ischemic Stroke: Looking Ahea,” Stroke 52 (2021): 3033. 34289710 10.1161/STROKEAHA.121.032241 PMC8384682

[2] Á. Chamorro , E. H. Lo , A. Renú , K. van Leye , and P. D. Lyden , “The Future of Neuroprotection in Stroke,” Journal of Neurology, Neurosurgery & Psychiatry 92 (2021): 129. 33148815 10.1136/jnnp-2020-324283

[3] E. Bandera , M. Botteri , C. Minelli , A. Sutton , K. R. Abrams , and N. Latronico , “Cerebral Blood Flow Threshold of Ischemic Penumbra and Infarct Core in Acute Ischemic Stroke: a Systematic Review,” Stroke 37 (2006): 1334. 16574919 10.1161/01.STR.0000217418.29609.22

[4] A. Brookshier and P. Lyden , “Differential Vulnerability among Cell Types in the Neurovascular Unit: Description and Mechanisms,” Journal of Cerebral Blood Flow & Metabolism 45 (2025): 3. 39520113 10.1177/0271678X241299960 PMC11563522

[5] L. Catanese , J. Tarsia , and M. Fisher , “Acute Ischemic Stroke Therapy Overview,” Circulation Research 120 (2017): 541. 28154103 10.1161/CIRCRESAHA.116.309278

[6] T. D. Faizy , M. Mlynash , R. Kabiri , et al., “The Cerebral Collateral Cascade Comprehensive Blood Flow in Ischemic Stroke,” Neurology (2022): e2296–e2306. 35483902 10.1212/WNL.0000000000200340

[7] M. Rocha and T. G. Jovin , “Fast versus Slow Progressors of Infarct Growth in Large Vessel Occlusion Stroke,” Stroke; A Journal of Cerebral Circulation 48 (2017): 2621. 10.1161/STROKEAHA.117.017673 28794271

[8] L. R. Wechsler , O. Adeoye , F. Alemseged , et al., “Most Promising Approaches to Improve Stroke Outcomes: the Stroke Treatment Academic Industry Roundtable XII Workshop,” Stroke 54 (2023): 3202. 37886850 10.1161/STROKEAHA.123.044279

[9] Y. Xiong , B. Manwani , and M. Fisher , “Management of Acute Ischemic Stroke,” The American Journal of Medicine 132 (2019): 286–291. 30832769 10.1016/j.amjmed.2018.10.019

[10] J. Christenson , M. D. Hill , R. H. Swartz , et al., “Efficacy and Safety of Intravenous Nerinetide Initiated by Paramedics in the Field for Acute Cerebral Ischaemia within 3 h of Symptom Onset (FRONTIER): a Phase 2, Multicentre, Randomised, Double‐blind, Placebo‐controlled Study,” The Lancet 405 (2025): 571–582. 10.1016/S0140-6736(25)00193-X 39955120

[11] H. K. Eltzschig and T. Eckle , “Ischemia and Reperfusion—From Mechanism to Translation,” Nature Medicine 2011 (1391): 1391–1401. 10.1038/nm.2507 PMC3886192 22064429

[12] M. Duering , R. Righart , F. A. Wollenweber , V. Zietemann , B. Gesierich , and M. Dichgans , “Acute Infarcts Cause Focal Thinning in Remote Cortex via Degeneration of Connecting fiber Tracts,” Neurology 84 (2015): 1685–1692. 25809303 10.1212/WNL.0000000000001502 PMC4409580

[13] K. A. Jones , S. Maltby , M. W. Plank , et al., “Peripheral Immune Cells Infiltrate into Sites of Secondary Neurodegeneration after Ischemic Stroke,” Brain, Behavior, and Immunity 67 (2018): 299–307. 28911981 10.1016/j.bbi.2017.09.006

[14] G. Ma , R. Mo , X. Yao , et al., “Clinical and Safety Outcomes of Edaravone Dexborneol in Acute Ischemic Stroke A Multicenter, Prospective, Cohort Study,” Neurology (2025): 213949. 10.1212/WNL.0000000000213949 40763317

[15] Y. Fu , A. Wang , R. Tang , et al., “Sublingual Edaravone Dexborneol for the Treatment of Acute Ischemic Stroke,” JAMA Neurology 81 (2024): 319–326. 38372981 10.1001/jamaneurol.2023.5716 PMC10877503

[16] P. Lyden , K. E. Pryor , C. S. Coffey , et al., “Final Results of the RHAPSODY Trial: a Multi‐Center, Phase 2 Trial Using a Continual Reassessment Method to Determine the Safety and Tolerability of 3K3A‐APC, a Recombinant Variant of Human Activated Protein C, in Combination with Tissue Plasminogen Activator, Mechanical Thrombectomy or Both in Moderate to Severe Acute Ischemic Stroke,” Annals of Neurology 85 (2019): 125–136. 30450637 10.1002/ana.25383 PMC6342508

[17] V. Stanislaus , A. Kam , L. Murphy , et al., “A Feasibility and Safety Study of afamelanotide in Acute Stroke Patients – an Open Label, Proof of Concept, Phase iia Clinical Trial,” BMC Neurology 23 (2023): 281, 10.1186/s12883-023-03338-9. 37496004 PMC10373257

[18] M. Hernández‐Jiménez , F. Abad‐Santos , I. Cotgreave , et al., “Safety and Efficacy of ApTOLL in Patients with Ischemic Stroke Undergoing Endovascular Treatment,” JAMA Neurology 80 (2023): 779–788, 10.1001/jamaneurol.2023.1660. 37338893 PMC10282959

[19] L. M. Manna , J. Hernandez , D. E. Hunt , et al., “First‐in‐Human Phase I Clinical Trial of the Adenosine A1R/A3R Agonist AST‐004 in Healthy Subjects,” Stroke; A Journal of Cerebral Circulation 55 (2024): 2795. 10.1161/STROKEAHA.124.047207 39450498

[20] D. R. Khasanova and M. N. Kalinin , “Cerebrolysin as an Early Add‐on to Reperfusion Therapy: Risk of Hemorrhagic Transformation after Ischemic Stroke (CEREHETIS), a Prospective, Randomized, Multicenter Pilot Study,” BMC Neurology 23 (2023): 121. 36973684 10.1186/s12883-023-03159-w PMC10041692

[21] Z. Poljakovic , S. Supe , J. Ljevak , et al., “Efficacy and Safety of Cerebrolysin after Futile Recanalisation Therapy in Patients with Severe Stroke,” Clinical Neurology and Neurosurgery 207 (2021): 106767. 34214867 10.1016/j.clineuro.2021.106767

[22] A. Agarwal , V. Y. Vishnu , J. Sharma , et al., “Citicoline in Acute Ischemic Stroke: a Randomized Controlled Trial,” PLoS ONE (2022). 10.1371/journal.pone.0269224 PMC9154187 35639720

[23] H.‐S. Chen , M.‐R. Chen , Y. Cui , et al., “Tenecteplase plus Butyphthalide for Stroke within 4.5–6 Hours of Onset (EXIT‐BT): a Phase 2 Study,” Translational Stroke Research 16 (2025): 575–583. 38238620 10.1007/s12975-024-01231-2

[24] A. Wang , B. Jia , X. Zhang , et al., “Efficacy and Safety of Butylphthalide in Patients with Acute Ischemic Stroke,” JAMA Neurology 80 (2023): 851–859. 37358859 10.1001/jamaneurol.2023.1871 PMC10294018

[25] R. Luo , R. Wangqin , L. Zhu , and W. Bi , “Neuroprotective Mechanisms of 3‑n‑Butylphthalide in Neurodegenerative Diseases (Review),” Biomedical Reports 11 (2019): 235–240. 31798868 10.3892/br.2019.1246 PMC6873419

[26] H.‐S. Chen , Z.‐A. Zhao , X.‐Y. Shen , et al., “Edaravone dexborneol for Ischemic Stroke with Sufficient Recanalization after Thrombectomy: a Randomized Phase II Trial,” Nature Communications 16 (2025): 2393. 10.1038/s41467-025-57774-x PMC11894225 40064868

[27] J. Xu , A. Wang , X. Meng , and G. Yalkun , et al., “Edaravone Dexborneol versus Edaravone Alone for the Treatment of Acute Ischemic Stroke: a Phase III, Randomized, Double‐Blind, Comparative Trial,” Stroke; A Journal of Cerebral Circulation 52 (2021): 772. 10.1161/STROKEAHA.120.031197 33588596

[28] J. Xu , Y. Wang , A. Wang , et al., “Safety and Efficacy of Edaravone Dexborneol versus edaravone for Patients with Acute Ischaemic Stroke: a Phase II, Multicentre, Randomised, Double‐blind, Multiple‐dose, Active‐controlled Clinical Trial,” Stroke and Vascular Neurology 4 (2019): 109. 31709115 10.1136/svn-2018-000221 PMC6812637

[29] R. Zhang , G. Liu , X. Zhao , et al., “Safety and Efficacy of GD‐11 in Patients with Ischaemic Stroke: a Multicentre, Double‐blind, Randomised, Placebo‐controlled, Phase 2 Trial,” Stroke Vascular Neurology 10 (2025): 003338, 10.1136/svn-2024-003338. PMC12107471 39107097

[30] L. Cai , Y. Ding , G. Rajah , et al., “Rapid Intravenous Glyceryl Trinitrate in Ischemic Damage (RIGID): A Potential Neuroprotection Strategy for Acute Ischemic Stroke (AIS) Patients,” Neurotherapeutics 21 (2024): 00365, 10.1016/j.neurot.2024.e00365. PMC11284536 38658264

[31] Z. Cheng , J. Gao , Y. Ding , Q. Pang , G. B. Rajah , and X. Geng , “Arterial Glyceryl Trinitrate in Acute Ischemic Stroke After Thrombectomy for Neuroprotection (AGAIN): A Pilot Randomized Controlled Trial,” Neurotherapeutics 20 (2023): 1746–1754. 37875733 10.1007/s13311-023-01432-x PMC10684471

[32] Q. Yang , C. Guo , C. Yue , et al., “Methylprednisolone as Adjunct to Endovascular Thrombectomy for Large‐Vessel Occlusion Stroke: The MARVEL Randomized Clinical Trial,” Jama 331 (2024): 840. 38329440 10.1001/jama.2024.0626 PMC10853866

[33] J. S. Lee , H. G. Kang , S. H. Ahn , et al., “Nelonemdaz and Patients with Acute Ischemic Stroke and Mechanical Reperfusion: The RODIN Randomized Clinical Trial,” JAMA Network Open 8 (2025): 2456535. 10.1001/jamanetworkopen.2024.56535 PMC11775734 39874036

[34] J. M. Hong , J. S. Lee , Y.‐B. Lee , et al., “Nelonemdaz for Patients With Acute Ischemic Stroke Undergoing Endovascular Reperfusion Therapy: A Randomized Phase II Trial,” Stroke 53 (2022): 3250. 36065810 10.1161/STROKEAHA.122.039649 PMC9586831

[35] M. D. Hill , M. Goyal , A. M. Demchuk , et al., “Efficacy and Safety of nerinetide in Acute Ischaemic Stroke in Patients Undergoing Endovascular Thrombectomy without Previous Thrombolysis (ESCAPE‐NEXT): a Multicentre, Double‐blind, Randomised Controlled Trial,” The Lancet 405 (2025): 560–570. 10.1016/S0140-6736(25)00194-1 39955119

[36] A.‐C. Chao , T.‐H. Lee , L. C. Pettigrew , et al., “Intravenous Odatroltide for Acute Ischemic Stroke within 24 Hours of Onset: a Phase 2, Multicenter, Randomized, Double‐Blind, Placebo‐Controlled Study,” Drug Design, Development and Therapy 18 (2024): 2033–2042. 38859883 10.2147/DDDT.S460831 PMC11164084

[37] J. S. Kim , K. B. Lee , J.‐H. Park , et al., “Safety and Efficacy of Otaplimastat in Patients with Acute Ischemic Stroke Requiring tPA (SAFE‐TPA): a Multicenter, Randomized, Double‐Blind, Placebo‐Controlled Phase 2 Study,” Annals of Neurology 87 (2020): 233–245. 31721277 10.1002/ana.25644 PMC7003891

[38] S. Ghosh , J. S. Dubow , J. Sutherland , et al., “Randomized, Proof‐of‐Concept Trial (RESCUE) of RNS60 as an Adjunct Therapy in Acute Ischemic Stroke,” Stroke; A Journal of Cerebral Circulation 56 (2025): 2386. 10.1161/STROKEAHA.125.051179 40671649

[39] K. M. Sicard and M. Fisher , “Animal Models of Focal Brain Ischemia,” Stroke Medicine 1 (2009): 7. 10.1186/2040-7378-1-7 PMC2820445 20150985

[40] E. Lorenzi , A. M. Crawford , C. S. Anderson , et al., “Adaptive Platform Trials in Stroke,” Stroke; A Journal of Cerebral Circulation 56 (2025): 198–208. 10.1161/STROKEAHA.124.045754 PMC11665893 39705391

[41] A. van der Meij , M. A. A. van Walderveen , N. D. Kruyt , et al., “NOn‐Invasive Vagus Nerve Stimulation in Acute Ischemic Stroke (NOVIS): a Study Protocol for a Randomized Clinical Trial,” Trials 21 (2020): 878. 33106174 10.1186/s13063-020-04794-1 PMC7586413

[42] R. A. Blauenfeldt , J. L. Waller , K. R. Drasbek , et al., “Effect of Remote Ischemic Conditioning and Red Blood Cells Biomarkers on Outcomes in Patients With Acute Stroke,” Journal of the American Heart Association 14 (2025): 040787. 10.1161/JAHA.124.040787 PMC12184582 40357654

[43] Z.‐N. Guo , R. Abuduxukuer , P. Zhang , et al., “Safety and Efficacy of Remote Ischemic Conditioning in Patients with Intravenous Thrombolysis: the SERIC‐IVT Trial,” Stroke; A Journal of Cerebral Circulation 56 (2025): 335. 10.1161/STROKEAHA.124.048509 39772709

[44] W. Li , S. Wang , L. Liu , et al., “Normobaric Hyperoxia Combined with Endovascular Treatment Based on Temporal Gradient: a Dose‐Escalation Study,” Stroke; A Journal of Cerebral Circulation 55 (2024): 1468. 10.1161/STROKEAHA.123.046106 38747162

[45] G. F. Cattaneo , A. M. Herrmann , S. A. Eiden , et al., “Selective Intra‐carotid Blood Cooling in Acute Ischemic Stroke: a Safety and Feasibility Study in an Ovine Stroke Model,” Journal of Cerebral Blood Flow & Metabolism 41 (2021): 3097. 34159825 10.1177/0271678X211024952 PMC8756475

[46] Y. Xu , W. Hu , X. Chen , et al. Stroke; A Journal of Cerebral Circulation 56 (2025): 240.

[47] C. Ayata , P. M. Bath , A. M. Planas , et al. Stroke; A Journal of Cerebral Circulation (2025).

[48] M. Fisher and S. I. Savitz , “Pharmacological Brain Cytoprotection in Acute Ischaemic Stroke — Renewed Hope in the Reperfusion Era,” Nature Reviews Neurology 18 (2022): 193–202. 35079135 10.1038/s41582-021-00605-6 PMC8788909

[49] B. A. Sutherland , A. A. Neuhaus , Y. Couch , et al., “The Transient Intraluminal Filament Middle Cerebral Artery Occlusion Model as a Model of Endovascular Thrombectomy in Stroke,” Journal of Cerebral Blood Flow & Metabolism 36 (2016): 363. 26661175 10.1177/0271678X15606722 PMC4759672

[50] S. Yoshimura , M. Dorok , U. Mamrak , et al., “Reliable Infarction of the Middle Cerebral Artery Territory in C57BL/6 Mice Using Pterygopalatine Artery Ligation and Filament Optimization – The PURE‐MCAo Model,” Journal of Cerebral Blood Flow & Metabolism 45 (2025): 871. 39370987 10.1177/0271678X241281841 PMC11563556

[51] Y. Chen , W. Zhu , W. Zhang , et al., “A Novel Mouse Model of Thromboembolic Stroke,” Journal of Neuroscience Methods 256 (2015): 203–211. 26386284 10.1016/j.jneumeth.2015.09.013 PMC4651806

[52] L. Zhang , R. L. Zhang , Q. Jiang , G. Ding , M. Chopp , and Z. G. Zhang , “Focal Embolic Cerebral Ischemia in the Rat,” Nature Protocols 10 (2015): 539–547. 25741989 10.1038/nprot.2015.036 PMC4602402

[53] I.‐E. Mosneag , S. M. Flaherty , R. C. Wykes , and S. M. Allan , “Stroke and Translational Research – Review of Experimental Models With a Focus on Awake Ischaemic Induction and Anaesthesia,” Neuroscience 550 (2024): 89–101. 38065289 10.1016/j.neuroscience.2023.11.034

[54] A. B. Uzdensky , “Photothrombotic Stroke as a Model of Ischemic Stroke,” Translational Stroke Research 9 (2018): 437–451. 29188434 10.1007/s12975-017-0593-8

[55] D. Kaiser , G. Weise , K. Möller , et al., “Spontaneous White Matter Damage, Cognitive Decline and Neuroinflammation in Middle‐aged Hypertensive Rats: an Animal Model of Early‐stage Cerebral Small Vessel Disease,” Acta Neuropathologica Communications 2 (2014): 169. 25519173 10.1186/s40478-014-0169-8 PMC4279586

[56] M. J. Cipolla and S.‐L. Chan , “Impact of Acute and Chronic Hypertension on Changes in Pial Collateral Tone in Vivo during Transient Ischemia,” Hypertension 76 (2020): 1019. 32683904 10.1161/HYPERTENSIONAHA.120.15356 PMC7429292

[57] A. Letourneur , S. Roussel , J. Toutain , M. Bernaudin , and O. Touzani , “Impact of Genetic and Renovascular Chronic Arterial Hypertension on the Acute Spatiotemporal Evolution of the Ischemic Penumbra: a Sequential Study with MRI in the Rat,” Journal of Cerebral Blood Flow & Metabolism 31 (2011): 504. 20648035 10.1038/jcbfm.2010.118 PMC3049506

[58] D. H. Lau , N. J. Shipp , D. J. Kelly , et al. PLoS ONE 8 (2013): 72416. 10.1371/journal.pone.0072416 PMC3754972 24013508

[59] L. J. Dalco and K. R. Dave Methods in Molecular Biology 2616 (2023): 2616. 10.1007/978-1-0716-2926-0_30 36715951

[60] J. Chen , X. Cui , A. Zacharek , Y. Cui , C. Roberts , and M. Chopp , Stroke 42 (2011): 445. 21193743 10.1161/STROKEAHA.110.596486 PMC3108495

[61] M. J. Haley , S. Krishnan , D. Burrows , et al., “Acute High‐Fat Feeding Leads to Disruptions in Glucose Homeostasis and Worsens Stroke Outcome,” Journal of Cerebral Blood Flow & Metabolism 39 (2019): 1026. 29171775 10.1177/0271678X17744718 PMC6545621

[62] C. Ayata , H. K. Shin , E. Dileköz , et al., “Hyperlipidemia Disrupts Cerebrovascular Reflexes and Worsens Ischemic Perfusion Defect,” Journal of Cerebral Blood Flow & Metabolism 33 (2013): 954–962. 23486293 10.1038/jcbfm.2013.38 PMC3677117

[63] T.‐H. Kim and R. Vemuganti , “Effect of Sex and Age Interactions on Functional Outcome after Stroke,” CNS Neuroscience & Therapeutics 21 (2015): 327–336. 25404174 10.1111/cns.12346 PMC6495347

[64] L. L. Cui , D. Golubczyk , A. M. Tolppanen , J. Boltze , and J. Jolkkonen , “Cell Therapy for Ischemic Stroke: ,Are Differences in Preclinical and Clinical Study Design Responsible for the Translational Loss of Efficacy?,” Annals of Neurology 86 (2019): 5, 10.1002/ana.25493. 31020699

[65] J. Boltze , B. Lukomska , J. Jolkkonen J ;, and M.‐I. consortium , “Mesenchymal Stromal Cells in Stroke: Improvement of Motor Recovery or Functional Compensation?,” Journal of Cerebral Blood Flow & Metabolism 34 (2014): 1420–1421. 24849662 10.1038/jcbfm.2014.94 PMC4126100

[66] X. Z. Shen , Y. Li , L. Li , et al., “Microglia Participate in Neurogenic Regulation of Hypertension,” Hypertension 66 (2015): 309–316. 26056339 10.1161/HYPERTENSIONAHA.115.05333 PMC4498964

[67] L. Marks , H. V. Carswell , E. E. Peters , et al., “Characterization of the Microglial Response to Cerebral Ischemia in the Stroke‐Prone Spontaneously Hypertensive Rat,” Hypertension 38 (2001): 116–122, 10.1161/01.HYP.38.1.116. 11463771

[68] K. Möller , C. Pösel , A. Kranz , et al., Frontiers in Cellular Neuroscience 9 (2015): 461. 26640428 10.3389/fncel.2015.00461 PMC4661280

[69] M. M. Santisteban , N. Ahmari , J. M. Carvajal , et al., “Involvement of Bone Marrow Cells and Neuroinflammation in Hypertension,” Circulation Research 117 (2015): 178–191. 25963715 10.1161/CIRCRESAHA.117.305853 PMC4490954

[70] S. Chrissobolis , A. A. Miller , G. R. Drummond , B. K. Kemp‐Harper , and C. G. Sobey , “Oxidative Stress and Endothelial Dysfunction in Cerebrovascular Disease,” Frontiers in Bioscience 16 (2011): 1733. 10.2741/3816 21196259

[71] P. Venkat , M. Chopp , and J. Chen , “Blood‐Brain Barrier Disruption, Vascular Impairment, and Ischemia/Reperfusion Damage in Diabetic Stroke,” Heart Assoc 6 (2017): 005819. 10.1161/JAHA.117.005819 PMC5669184 28572280

[72] L. Ritter , L. Davidson , M. Henry , et al., “Exaggerated Neutrophil‐Mediated Reperfusion Injury after Ischemic Stroke in a Rodent Model of Type 2 Diabetes,” Microcirculation 18 (2011): 552–561. 21699626 10.1111/j.1549-8719.2011.00115.x PMC8638747

[73] M. M. Elgebaly , S. Ogbi , W. Li , et al., “Neurovascular Injury in Acute Hyperglycemia and Diabetes: a Comparative Analysis in Experimental Stroke,” Translational Stroke Research 2 (2011): 391–398. 21909340 10.1007/s12975-011-0083-3 PMC3169178

[74] A. Ergul , M. Abdelsaid , A. Y. Fouda , and S. C. Fagan , “Cerebral Neovascularization in Diabetes: Implications for Stroke Recovery and beyond,” Journal of Cerebral Blood Flow & Metabolism 34 (2014): 553–563. 24496174 10.1038/jcbfm.2014.18 PMC3982092

[75] D. M. Hermann , T. R. Doeppner , and A. Popa‐Wagner , “Opportunities and Limitations of Vascular Risk Factor Models in Studying Plasticity‐Promoting and Restorative Ischemic Stroke Therapies,” Neural Plasticity 2019 (2019): 1. 10.1155/2019/9785476 PMC6885287 31827502

[76] M. Ishikawa , K. Y. Stokes , J. H. Zhang , A. Nanda , and D. N. Granger , “Role of P‐selectin in Leukocyte Recruitment and Brain Injury After Focal Cerebral Ischemia and Reperfusion,” Circulation Research 94 (2004): 239. 14670846 10.1161/01.RES.0000111524.05779.60

[77] J. Herz , P. Sabellek , T. E. Lane , M. Gunzer , D. M. Hermann , and T. R. Doeppner , “Role of Neutrophils in Exacerbation of Brain Injury after Focal Cerebral Ischemia in Hyperlipidemic Mice,” Stroke; A Journal of Cerebral Circulation 46 (2015): 2916–2925. 10.1161/STROKEAHA.115.010620 PMC4589522 26337969

[78] X.‐L. Cao , J. Du , Y. Zhang , J.‐T. Yan , and X.‐M. Hu , “Hyperlipidemia Exacerbates Cerebral Injury through Oxidative Stress, Inflammation and Neuronal Apoptosis in MCAO/Reperfusion Rats,” Experimental Brain Research 233 (2015): 2753–2765. 26238404 10.1007/s00221-015-4269-x

[79] J. Deng , J. Zhang , C. Feng , L. Xiong , and Z. Zuo , “Critical Role of Matrix Metalloprotease‐9 in Chronic High Fat Diet‐induced Cerebral Vascular Remodelling and Increase of Ischaemic Brain Injury in Mice,” Cardiovascular Research 103 (2014): 473–484. 24935427 10.1093/cvr/cvu154 PMC4200053

[80] M. J. Haley , G. Mullard , K. A. Hollywood , G. J. Cooper , W. B. Dunn , and C. B. Lawrence , “Adipose tissue and metabolic and inflammatory responses to stroke are altered in obese mice,” Disease Models & Mechanisms 10 (2017): 1229–1243. 28798136 10.1242/dmm.030411 PMC5665457

[81] X. Li , H. Guo , L. Zhao , et al. Biochimica Et Biophysica Acta, Molecular Basis of Disease 2017 (1863): 3265. 10.1016/j.bbadis.2017.08.010 28844957

[82] S. Li and M. Fisher Stroke; A Journal of Cerebral Circulation 54 (2023): e16–e19. 10.1161/STROKEAHA.122.041354 36503265

[83] A. Taha , J. Bobi , R. Dammers , et al., “Comparison of Large Animal Models for Acute Ischemic Stroke: which Model to Use?,” Stroke; A Journal of Cerebral Circulation 53 (2022): 1411–1422. 10.1161/STROKEAHA.121.036050 PMC10962757 35164533

[84] O. W. Brooks , R. M. King , E. Nossek , et al., “A Canine Model of Mechanical Thrombectomy in Stroke,” Journal of NeuroInterventional Surgery 11 (2019): 1243. 31103992 10.1136/neurintsurg-2019-014969

[85] J. Boltze , F. Nitzsche , J. Jolkkonen , et al., “Concise Review: Increasing the Validity of Cerebrovascular Disease Models and Experimental Methods for Translational Stem Cell Research,” Stem Cells 35 (2017): 1141–1153. 28207204 10.1002/stem.2595

[86] T. E. Liston , A. Hama , J. Boltze , et al., “Adenosine A1R/A3R (Adenosine A1 and A3 Receptor) Agonist AST‐004 Reduces Brain Infarction in a Nonhuman Primate Model of Stroke,” Stroke; A Journal of Cerebral Circulation 53 (2022): 238–248. 10.1161/STROKEAHA.121.036396 34802248

[87] P. Werner , D. Saur , V. Zeisig , et al., “Simultaneous PET/Mri in Stroke: a Case Series,” Journal of Cerebral Blood Flow & Metabolism 35 (2015): 1421–1425. 26174332 10.1038/jcbfm.2015.158 PMC4640332

[88] M. S. Shazeeb , R. M. King , O. W. Brooks , et al., “Infarct Evolution in a Large Animal Model of Middle Cerebral Artery Occlusion,” Translational Stroke Research 11 (2020): 468–480. 31478129 10.1007/s12975-019-00732-9 PMC7051891

[89] A. M. Herrmann , S. Meckel , M. J. Gounis , et al., “Large Animals in Neurointerventional Research: a Systematic Review on Models, Techniques and Their Application in Endovascular Procedures for Stroke, Aneurysms and Vascular Malformations,” Journal of Cerebral Blood Flow & Metabolism 39 (2019): 375–394. 30732549 10.1177/0271678X19827446 PMC6421248

[90] S. I. Savitz and J.‐C. Baron , “STAIR X Consortium,” M Fisher 3 (2019): 1026.

[91] S. Rehman , A. Nadeem , A. B. Ud Kasi , et al., AJNR Am J Neuroradiol 2025, 46, 1069. 39477546 10.3174/ajnr.A8557 PMC12152793

[92] R. W. Regenhardt , C. A. Potter , S. S. Huang , and M. H. Lev , “Advanced Imaging for Acute Stroke Treatment Selection,” Radiologic Clinics of North America 61 (2023): 445–465. 36931761 10.1016/j.rcl.2023.01.003

[93] M. Olivé‐Gadea , M. Requena , D. Campos , et al., “Defining a Target Population to Effectively Test a Neuroprotective Drug,” Stroke; A Journal of Cerebral Circulation 52 (2021): 505–510. 10.1161/STROKEAHA.120.032025 33423513

[94] E. A. Samaniego , J. Boltze , P. D. Lyden , et al., “Priorities for Advancements in Neuroimaging in the Diagnostic Workup of Acute Stroke.,” Stroke 54 (2023): 3190–3201. 37942645 10.1161/STROKEAHA.123.044985 PMC10841844

[95] J. P. Broderick , E. A. Mistry , P. M. Wechsler , et al., “Stroke Treatment Academic Industry Roundtable XIII Participants,” Stroke n/a (2025).

[96] J. L. Saver , N. Chaisinanunkul , B. C. V. Campbell , et al., “XIth Stroke Treatment Academic Industry Roundtable,” Stroke 52 (2021): 3054. 34320814 10.1161/STROKEAHA.121.034480

[97] J. Bai and P. D. Lyden , “Revisiting Cerebral Postischemic Reperfusion Injury: New Insights in Understanding Reperfusion Failure, Hemorrhage, and Edema,” International Journal of Stroke 10 (2015): 143–152. 25598025 10.1111/ijs.12434

[98] X. Nie , X. Leng , Z. Miao , M. Fisher , and L. Liu , “Clinically Ineffective Reperfusion After Endovascular Therapy in Acute Ischemic Stroke,” Stroke; A Journal of Cerebral Circulation 54 (2023): 873–881. 10.1161/STROKEAHA.122.038466 36475464

[99] F. Pariona‐Vargas , K. T. Mun , E. H. Lo , et al., “Is There Diurnal Variation in Magnesium Neuroprotective and Thrombolytic Therapy Effect Upon Acute Cerebral Ischemia Outcome?,” Journal of Stroke and Cerebrovascular Diseases 34 (2025): 108278. 40054793 10.1016/j.jstrokecerebrovasdis.2025.108278

[100] P. Rajput , A. Brookshier , S. Kothari , et al. Journal of Neuroscience 44 (2024):1093222024. 10.1523/JNEUROSCI.1093-22.2024 PMC11140689 38548341

